# 临床病理讨论-双肺弥漫性间质性疾病

**DOI:** 10.3779/j.issn.1009-3419.2011.01.15

**Published:** 2011-01-20

**Authors:** 恒娟 呙, 林娟 连, 殿胜 钟

**Affiliations:** 300052 天津，天津医科大学总医院呼吸内科 Department of Respiratory Medicine, Tianjin Medical University General Hospital, Tianjin 300052, China

## 1 临床资料

患者，女，75岁，主因“间断咳嗽1年余，活动后气促1月”于2010年9月2日收住我院。入院前1年无明显诱因出现咳嗽、少痰，多为刺激性干咳，无明显季节性，夜间可平卧，间断服用镇咳药物治疗，症状无明显缓解。近1月来出现活动后气促，活动耐力较前明显下降，遂到我院就诊，胸部CT显示：两肺多发异常通亮区，以两上叶为著，右下叶实变影，双肺间质纹理增多、紊乱，双下肺呈蜂窝状改变趋向，纵膈（气管前间隙、隆突下间隙）可见肿大淋巴结影，考虑“双肺气肿、双肺间质性病变、纤维化”。为求进一步诊治收入院。自发病以来，精神、食欲、睡眠可，二便如常，体重无著变。

既往体健，否认高血压、冠心病、糖尿病、慢性呼吸系统疾病和结核史，否认皮疹和骨关节慢性疼痛史。吸烟60年，平均20支/日。否认毒物接触和养宠物、家禽史，否认粉尘接触史，无长期服药史。

查体：体温36.5 ℃，脉搏80次/分，呼吸20次/分，血压135 mmHg/70 mmHg。神清合作，营养中等，全身皮肤粘膜无黄染，口唇无紫绀，浅表淋巴结未触及肿大，颈静脉无怒张，胸廓对称，双下肺可闻及散在Velcro啰音，心脏相对浊音界不大，心律齐，各瓣膜听诊区未闻及杂音，腹软，无压痛及反跳痛，肝脾肋下未触及；双下肢无水肿，无杵状指（趾）。

入院诊断：双肺弥漫性间质性疾病。

诊疗过程：入院后完善相关检查。血常规：WBC 7.58×10^9^/L，N 58.7%，Hb 104 g/L，PLT 386×10^9^/L；血气分析（不吸氧、安静状态下）：pH7.458，PCO_2_ 38.5 mmHg，PO_2_ 64.7 mmHg；肝肾功能和血糖正常。肺功能：一氧化碳弥散量（diffusing capacity of the lung for carbon monoxide, DLCO）45%，用力肺活量（forced vital capacity, FVC）65%，一秒钟用力呼气量（forced expiratory volume in first second, FEV_1_）75%，FEV_1_/FVC 89%，符合轻度限制性通气功能障碍伴中度弥散功能减退。胸部高分辨率CT（high resolution computerized tomography, HRCT）：双上肺气肿；两肺间质炎症和间质纤维化改变，以两下肺外周区为著，考虑寻常型间质性肺炎（usual interstitial pneumonia, UIP）可能性大（图 1）。血清免疫学检查：IgG、IgA、IgM正常，风湿抗体（extractable nuclear antigen, ENA）、抗核抗体（antinuclear antibody, ANA）和抗中性粒细胞胞浆抗体（antineutrophil cytoplasmic antibodies, ANCA）等均阴性，C反应蛋白（C-reactive protein, CRP）2.6 mg/dL（正常 < 0.86 mg/dL），类风湿因子（rheumatoid factor, RF）124 IU/mL（正常 < 20 IU/mL）。类风湿五项：抗RA33抗体158.4 U/mL（正常 < 25 U/mL），抗环瓜氨酸肽抗体（anti-cyclic citrullinated peptide antibody, CCPAb）100.8 U/mL（正常 < 12 U/mL），RF-IgA 1.2 U/mL（正常 < 12 U/mL），RF-IgG 298.6 U/mL（正常 < 12 U/ mL），抗角蛋白抗体（antikeratin antibody, AKA）为阳性。纤维支气管镜检查：慢性支气管炎症；支气管肺泡灌洗液：肺泡巨噬细胞19%，淋巴细胞5%，中性粒细胞75.5%，嗜酸性粒细胞0.5%；右下叶外侧基底段纤维支气管镜肺活检（transbronchial lung biopsy, TBLB）病理：小细胞恶性肿瘤，首先考虑为小细胞肺癌（图 2）。CT引导下经皮右下肺穿刺活检病理诊断：小细胞肺癌（图 3）。免疫组化染色：CK、CgA和Syn阳性，34βE12、TTF-1和LCA阴性。

**1:**
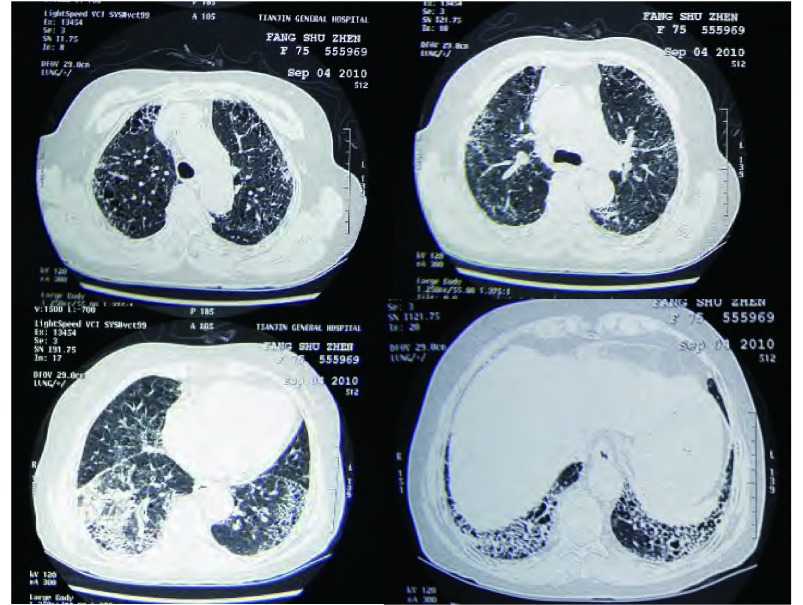
胸部HRCT：双上肺气肿；两肺间质炎症和间质纤维化改变，以两下肺外周区为著，呈蜂窝状改变，右下叶实变影。 HRCT of thorax: Emphysema existed in the upper lobes of the lungs, consolidation in the right lower lobe, marked coarse reticular opacities, traction bronchiecatsis, and honeycomb cyst in the basilar and peripheral (subpleural) regions of the lungs.

**2:**
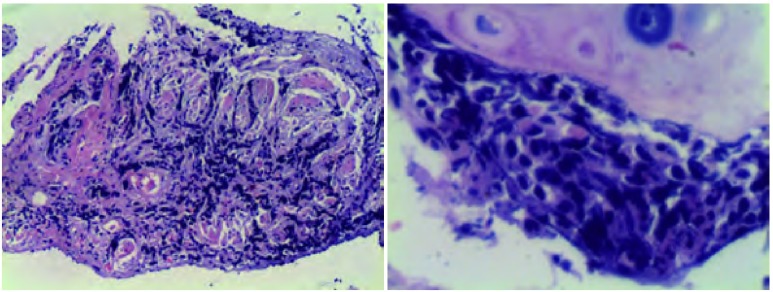
经支气管镜肺活检（右下叶外侧基底段），小细胞恶性肿瘤，首先考虑为小细胞肺癌。 The pathological type of transbronchial lung biopsy (TBLB) showed small cell carcinoma, small cell lung cancer (SCLC) was considered first.

**3:**
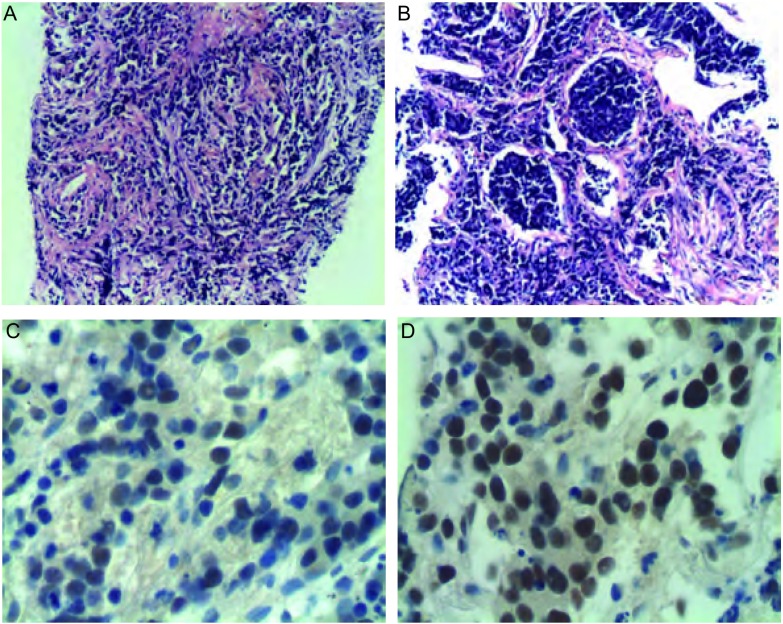
经皮肺穿病理诊断为小细胞肺癌。A：低倍镜下视野；B：高倍镜下表现：可见癌细胞巢；C：CgA免疫组化染色阳性；D：Syn免疫组化染色阳性。 The pathological diagnosis of CT-guided transthoracic needle biopsy was SCLC. A: Low-power field of microscope; B: Cancer cells nests were observed under high-power field of microscope; C: Immunohistochemisty (IHC) staining of CgA showed positive result; D: IHC result of Syn was positive.

## 2 点评

钟殿胜医师：该患者，老年女性，临床表现以咳嗽，活动后气促为主，查体双肺可闻及Velcro音，肺功能符合轻度限制性通气功能障碍伴中度弥散功能减退。胸部CT，尤其是HRCT显示两肺多发异常通亮区，以两上叶为著；双肺间质纹理增多、紊乱，两肺间质炎症和间质纤维化改变，以两下肺外周区为著，呈蜂窝状改变；此外，右下叶可见实变影。上述特点提示，患者为一典型的双肺弥漫性疾病，以肺间质改变为主，伴有部分实变，但较少磨玻璃状阴影。弥漫性间质性肺疾病（interstitial lung disease, ILD）是一组异质性的肺部非肿瘤性疾病，在病理学上表现为不同类型的间质性肺炎和纤维化，目前已有200多种疾病囊括在ILD之下，而且大多数病变缺乏特征性，其诊断是临床医师常面临的一个棘手的问题。根据2002年美国胸科学会（American Thoracic Society, ATS）和欧洲呼吸协会（European Respiratory Society, ERS）达成的共识，弥漫性ILD可以分为4大部分，即：有相关病因的ILD；特发性肺间质性肺炎（idiopathic interstitial pneumonia, IIP）；肉芽肿性疾患（如结节病）和特殊类型的ILD（如肺淋巴管平滑肌肌瘤病等）。在ATS/ERS分类和诊断标准中，强调了对IIP进行临床-影像-病理（clinico-radiologic-pathologic, CRP）诊断的重要性。放射科根据该患者HRCT表现考虑UIP的可能性大，但仅靠影像学不能做出IIP-UIP的诊断，必须结合临床。根据本患者的疾病特点及详细的问诊，临床上基本上可以除外感染因素、药物及职业环境等因素所导致的ILD。免疫全项、ENA和ANCA等化验正常，但类风湿检查中，抗R33抗体、抗环瓜氨酸肽抗体CCP-Ab、RF-IgG均明显高于正常，抗角蛋白抗体AKA阳性，虽然患者目前无相应的骨关节症状，但仍不能完全除外类风湿性关节炎（rheumatoid arthritis, RA）的可能，因为少部分RA患者，肺部受累可早于典型的关节炎出现，而RA所致肺损伤的临床表现与IIP并无区别，典型的影像学改变为双肺下叶基底段和胸膜下病变为重，病理类型以UIP为主。为了进一步明确诊断，我们给患者做了支气管镜-肺活检，病理结果提示为小细胞恶性肿瘤，首先考虑为小细胞肺癌。所以，我们接着做了CT引导下经皮右下肺穿刺活检，并加做了部分神经内分泌标志物的免疫组化染色，最终证实患者确系小细胞肺癌。

肺纤维化患者中肺癌的发病率明显高于普通人群，一般为9.8%-38%，组织学类型与一般肺癌的类型相似，以鳞癌和腺癌最为多见，占绝大多数，其它的包括小细胞癌、大细胞癌和混合癌等。肺间质纤维化伴发肺癌的患者，男性居多，发病年龄高于单纯肺间质纤维化患者平均年龄，平均年龄53岁-78岁，既往多有大量吸烟史；影像学检查兼具肺间质纤维化和肺癌两者的表现，绝大多数肿瘤位于外周，且多发生于纤维化程度较重的肺下叶，与蜂窝肺损害有密切关系，可表现为结节影、团块影，有时呈分叶状、有毛刺等。有些肺间质纤维化患者常可伴有感染，导致实变灶的形成，中晚期患者伴有纤维化瘢痕形成，在影像学上可掩盖肺肿瘤病灶，给诊断造成困难。该患者，老年女性，有长期吸烟史，临床表现、肺功能检查和HRCT检查提示为典型的双肺弥漫性间质性疾病，影像学上未见肿块和结节影，结合相关化验，首先考虑RA所致肺损伤，似乎诊断已经明确，但ILD的诊断强调了临床-影像-病理三者的结合，经TBLB应该属于常规检查，除了可以确诊某些疾病外，可起到除外其它疾病的鉴别诊断作用。该患者正是通过TBLB诊断为小细胞癌，并通过经皮肺穿刺进一步确诊为小细胞肺癌。考虑到小细胞肺癌的生长特性，影像上可以表现为非肿块性的弥漫性分布，该患者的确诊也证实了临床-影像-病理三者结合的重要性。

肺间质纤维化是肺癌的一个危险因素，有学者提出，对已明确诊断的特发性肺间质纤维化患者，当原有症状加重，特别是出现咯血、胸痛等，应警惕并发肺癌的可能性。对于肺间质纤维化与肺癌的关系以及两者并发的机制，目前仍不清楚，需要更进一步的研究。

